# Anthropocene angst: Authentic geology and stratigraphic sincerity

**DOI:** 10.1177/03063127241282309

**Published:** 2024-11-11

**Authors:** Alexander Damianos

**Affiliations:** Kent Law School, University of Kent, Canterbury, UK

**Keywords:** Anthropocene, angst, climate governance, authenticity, sincerity, normativity

## Abstract

In March 2024, the Anthropocene Working Group’s proposal for a formal Anthropocene Series/Epoch of the Geologic Time Scale was formally rejected by the Subcommission on Quaternary Stratigraphy. What does the failed formalization effort reveal about the relationship between science and normativity under conditions of ‘climate crisis’? Drawing on four years of ethnographic observation of the Anthropocene Working Group, this article explains how the Group developed its proposal, why it failed, and what it reveals about the social construction of geological truth. The effort to formalize an Anthropocene unit was based on a coupling of science and politics, wherein geo-scientists could make normative assertions in the register of scientific fact. Ultimately, the Group failed because it was seen as appropriating incumbent geological techniques to advance claims about the future, transitioning geology from a descriptive science about the past to a site of warning.

We do not live in the Anthropocene. This, at least, was the decision of the Subcommission on Quaternary Stratigraphy (SQS) of the International Commission on Stratigraphy (ICS), which arrived at that conclusion through a formal vote, the outcome of which was issued on the 5th of March, 2024. It came to this conclusion through a review of a proposal, submitted by the Anthropocene Working Group (AWG). The AWG was commissioned in 2009 by the SQS to determine whether the term ‘Anthropocene’, as promoted by Paul Crutzen and Eugene Stoermer (Crutzen, 2002; Crutzen & Stoermer, 2000), had merit as a formal unit of the Geologic Time Scale. It took the AWG almost fifteen years to come to the conclusion that, yes, the Anthropocene theme did merit consideration as a formal geological unit. The SQS, however, ultimately disagreed with the AWG’s reasoning. In addition to identifying material characteristics, such as traces of human activity that will obtain on a palaeontological register (as a technofossil, i.e. the fossil potential of the technosphere), the AWG also combed through previous decisions issued by the Subcommission to identify patterns in its decision-making and position the proposal accordingly. Though the SQS rejected the Anthropocene Series/Epoch proposal, the vote was not unanimous: two abstentions, four votes in favour, and twelve votes against. ‘The proposal,’ concludes the internal ballot receipt in large, underlined font, ‘not having received at least 60% of the YES votes, is not approved.’

This article is concerned with the consequences of the formalization effort and the manner in which geologists, and geo-scientists more generally, observe their own practice. The Anthropocene formalization effort was the result of attempts to articulate political and normative assertions on the register of geoscientific fact, due to a perceived gap between the severity of circumstances (all that falls under the umbrella term ‘climate crisis’) and the adequacy of responses from traditional normative avenues such as law and politics.

As generations of scholars have demonstrated, science is normative. What sets the Anthropocene formalization effort apart is that the term was first announced as an explicitly political statement. Only later did some geologists bring the term into the geosciences and commit themselves to its articulation as fact. Geoscientists both favourable and opposed to a formal Anthropocene Epoch/Series were aware of the essentially political valence of the term, even while working to articulate a scientific account of anthropogenic geological events. The Anthropocene, in other words, is symptomatic of emerging forms of climate governance beyond traditional avenues of politics, with angst becoming an *a priori* of scientific observation.

Many geo-scientists are folding angst into their research. Factual descriptions of phenomena ranging from global average temperatures to plutonium content of sedimentary deposits are framed within a narrative of concern. Some geoscientists accordingly position themselves not simply as passive conduits to policy decisions, nor even as ‘co-producers’ of regulatory decisions (Hulme, 2009; Jasanoff, 1994), but rather assume the duty of warning against future risk. In the case of the Anthropocene, techniques of measurement and observation are appropriated as strategies of warning against a vision of how society might unfold under conditions of climate change. These warnings are subsequently instrumentalized to justify the suggestion of extreme measures of counter-intervention (such as geo-engineering, of which Crutzen was an enthusiastic and outspoken proponent).

Throughout the fifteen years of the AWG’s formalization effort, the Group was repeatedly accused of conflating ‘science’ with ‘politics’ or ‘pop culture’ (Autin & Holbrook, 2012; Finney & Edwards, 2016). This has presented an unusual set of circumstances for geoscientific expertise, as peer-reviewed journal articles depart from discussion of rocks and sediments to an assessment of the intentionality of geoscientific research. The accusation that the AWG was primarily concerned not with ‘science’, but with performing what one interlocutor described as ‘big announcements to the press’, was one I came across frequently during time spent with geologists who had some scepticism about the AWG’s effort. Articles appearing in geoscientific journals often discuss the Anthropocene hypothesis in two registers: as a normative assertion and as a scientific truth claim (Autin & Holbrook, 2012; Edwards et al., 2022; Finney & Edwards, 2016; Gibbard et al., 2022; Zalasiewicz et al., 2021).

The effort to define an Anthropocene unit proceeded, and was ultimately assessed, not only with regard to the merit of the scientific research, but the conditions of geological observation. One could characterize the object of the AWG’s efforts to be the techniques, strategies, and conditions of administering standard classifications and nomenclature, rather than with classifying any particular unit. Insofar as the AWG’s proposal was ultimately decided through a vote, the conditions of geological observation were always already the primary object of inquiry. Yet there are legitimate reasons why the effort to formalize the Anthropocene is particularly illuminating, making it noteworthy for apprehending the normative valence of scientific research under conditions of ‘climate crisis’. Can geo-science contribute to steering a wider societal response to climate change? I argue that this is precisely what proponents of the Anthropocene Series/Epoch believed they could do. I will show how they sought to steer such a response by appropriating techniques of geological observation (palaeontology, chronostratigraphy and geochronology) to articulate a warning that would provoke a sense of urgency, as a proxy for asserting a normative claim on the register of geo-scientific fact.

## Observing geological observation

What would the Anthropocene have accomplished, had it been approved? First of all, it is worth pointing out the peculiar dynamics according to which the proposal for a formal Anthropocene Series/Epoch of the Geologic Time Scale was rejected. I have spoken to several members of the SQS in the run up to the AWG’s bid. They acknowledge that the AWG put together a sound proposal from a scientific perspective. By ‘scientific perspective’, they mean that the proposal included extremely detailed accounts of rock sections, sampled from across the Northern and Southern hemispheres and analysed using techniques ranging from palaeontological to radio-isotopic. However, the SQS is not a purely scientific organization, in the sense that it does not simply verify empirical scientific truth-claims. It administers geological time and space by proxy, by standardizing classificatory mechanisms and nomenclature applied to the past 2.58 million years of sedimentary accumulation on Earth. The administration of geological time requires more than scientific verification. It is also an ongoing exercise in epistemic governance. The SQS’s task is constituted through interpretation of particular presentations of evidence in such a way as to enforce normative practices of characterizing, defining, and standardizing the relationship between planetary time and space.

The AWG drew on precedent from previous SQS decisions regarding proposed changes to the Geologic Time Scale, seeking to frame and word their proposal similarly to earlier proposals that had been approved by the SQS. Certain methods are favoured in the SQS’s deliberative process. Palaeontology, the branch of stratigraphy that deals with making sense of fossil remains, entails soliciting testimony from sediments and traces, verified against the ‘facts of the matter’ put forward as the basis for the proposed amendment to the Geologic Time Scale, which is ultimately approved or rejected by a committee that possess an authority on the basis of presumed neutrality and soundness of judgement. The AWG, accordingly, sought to support its proposal by soliciting witness testimonies from fossils and sediments to develop a claim, which the SQS decision-making panel would approve or reject accordingly.

On the one hand, the AWG’s effort entailed the establishment of particular objects or historical events as geoscientific facts. Material remnants of human activity, ranging from plastic bottles to discarded chicken bones, were cast on the register of geo-history: the palaeontological record that will appear as of obvious geological significance on the level of other Series/Epochs (Bennett et al., 2018; Ivar do Sul & Labrenz, 2022; Williams et al., 2016). Consider a case that the AWG often cites in its advocacy of an Anthropocene Series/Epoch: Dinosaurs were wiped out by a meteorite strike that sprinkled Earth’s surface with iridium, an element that appears practically nowhere else on Earth prior to that impact event. Nuclear weapons detonation has covered Earth in a blanket of plutonium, specifically, ^239/240^Pu, otherwise incredibly rare in Earth’s rock record. ^239/240^Pu, in other words, provides a material correlate of a particular episode in human history. It allows the AWG to pattern its proposal as consistent with the decision making procedures that have characterized the formalization of other units of the Geologic Time Scale—the appearance of iridium being a key component in establishing the Cretaceous-Paleogene boundary (Zalasiewicz et al., 2019, pp. 282–284). As Pottage (2019) has noted, what makes the Anthropocene unusual is that ‘instead of beginning with the fossil and eliciting context from it, one begins with context and finds the Leitfossil for that context’ (Pottage, 2019, p. 154). The labour of the AWG entailed a process of realizing media that could help them narrate a historical account of human impact on global sedimentary accumulation.

For the AWG, unit formalization unfolded as a textual practice: writing papers that presented arguments in defence of a particular way of conducting geological observation. One unlikely benefit of the AWG receiving no financial support from any natural sciences funding organization^1^ is that it spent more time studying the papers associated with previous unit formalization proposals than it did studying sedimentary deposits in the field. This is evidenced by the fact that the AWG was commissioned in 2009, but did not have resources to conduct any of its own Anthropocene-specific field research until financing was secured from the Haus der Kulturen der Welt in 2018 (Rosol et al., 2023; Waters et al., 2023). The AWG submitted its proposal to the SQS five years later, in 2023. As the fact of the majority vote process indicates, the decision-making committee needed to be persuaded by specific practices of determining the ‘relevant factors’ in the constitution of a procedurally consistent narrative about the relation and administration of planetary time and space.

Voting members with whom I spoke in the weeks leading up to the AWG’s submission made clear that while they did not necessarily have a problem with, for example, radio-isotopic analysis of samples that indicated an exponential increase in ^239/240^Pu from samples across the world, between the years 1945 and 2000 (which is a miniscule duration in the context of 4.5 billion years of Earth history), they were very sceptical about granting Epoch/Series status (thereby ending the Holocene) to a unit that might be of such a small duration as to be less than the margin of error on geological units preceding it. Procedural constraints and expectations shaped the choice of signals observed as significant by the AWG (Head, Zalasiewicz et al., 2023; Zalasiewicz et al., 2017).

AWG members were always acutely aware of the dispositions of each member of the decision-making committee that ultimately rejected their proposal. Two members of the AWG, including its former Chair, Jan Zalasiewicz, and Martin Head, were also members of the decision-making committee. They were forbidden from voting on the final proposal, due to a perceived conflict of interest. The AWG sought to present their research in a way that would encourage a favourable response from the decision-making committee. During my time observing the AWG, one sentiment often repeated was of finding an analogous geological boundary, or previous decision of the ICS subgroups, to which the AWG could compare their proposed Anthropocene unit. For example, in comparing the Cretaceous-Paleogene boundary to the proposed Holocene-Anthropocene boundary, the AWG hoped to undermine any attempt to reject the Anthropocene proposal. Their approach mirrored legal practices of drawing on existing precedent to frame the facts of a case to provoke approval on procedural grounds. If the Anthropocene was *scientifically anomalous*, it might at least be *procedurally consistent*.

Above all, this strategy was pursued through an appropriation of palaeontological techniques from a mode of analysis that normally affords geologists access to deep time. The AWG’s effort suggests Kittler’s assertion that ‘media determine our situation’ has geo-scientific (or more specifically, chronostratigraphic) significance (Kittler, 1999, p. xxxix). Indeed, the generative potential of the Anthropocene theme emerges from the problem of articulating what would be determined, how geological insight facilitated social observation, and how social observation was folded into geo-administrative procedure. To appreciate how the Anthropocene Working Group developed a speculative palaeontological approach, I briefly outline the significance of the fossil as a discursive technique.

Beginning in the 17th century—and by some accounts, far earlier (Mayor, 2001)—strategies of narrating planetary dynamics become consolidated through material evidence, specifically fossils. Nicolas Steno’s discovery of shark teeth on top of the mountains of Tuscany are illustrative. Scripture provides an initial explanation: The teeth rose into a ‘great diluvial soup’ by the Great Flood, recounted in the Book of Genesis, subsequently re-deposited in unusual places. Yet if the teeth were found in rock, then they must be even older than the rock itself. For Steno, the discovery of stone-encrusted shark teeth on Italian mountains articulated a historiography far more complex than what the Bible could account for. Artefacts, evidently, possessed a chronology of their own. Human civilization was not alone in possessing a history. Steno’s findings echoed similar discoveries in other parts of Europe, including by Robert Hooke at the Royal Society, and the German Jesuit scholar Athanasius Kircher (Gould, 1987; Rappaport, 1986; Rudwick, 2017, pp. 9–30).

**Figure 1.**
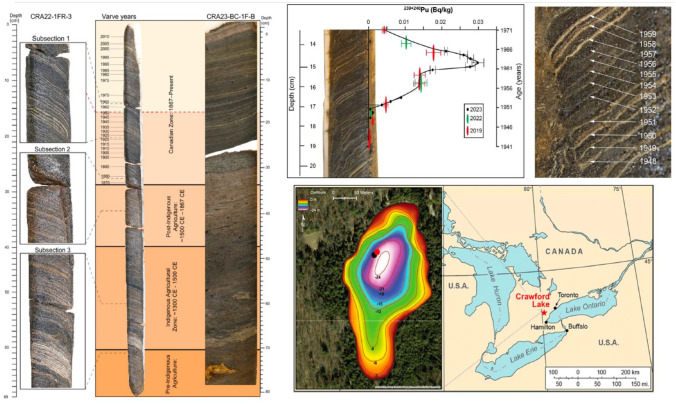
The core that provided the basis of the Anthropocene Working Group’s claim for a geologically valid ‘lower boundary’, or stratigraphic ‘beginning’ of their proposed Anthropocene Series/Epoch, together with a map of where the core was extracted from. Layers of the core can be dated annually. Once chemically analysed, it was found that levels of ^239/240^Pu begin a noticeable onset at the layer that is dated from 1952. Source: McCarthy et al. (2023).

Here one sees the initial forensic gesture. The premise that a new category of artefact, ‘natural antiquities’, could recount a history of the planet as distinct from human history, was a subtle yet radical gesture. The fossil became a jurisprudential technique, submitting the material environment to scrutiny, implying ‘a gesture that in a juridical context would be characterized as the designation of a responsible agency’ (Sloterdijk, 2018). Scripture provided an account of the planet, a way of verifying the material environment; with the advent of fossils, the material environment became a technique of verifying scripture. This strategy was subsequently extrapolated such that geologists come to position themselves as intermediaries of geological deep time. Through palaeontological practices, they speak on behalf of fossils, rocks, and sediments, even though they remain separated from the events recounted in their testimony. In soliciting testimony from fossils, geologists generate novel insights into the material and epistemic constitution of Earth itself (Figure 1).

In the context of the Anthropocene, the process of observing material characteristics to piece together a discursive practice called ‘geology’ becomes itself geologically observable. Members of the AWG proposed the term ‘technofossil’ to emphasize the novelty of post-WWII sediments (Zalasiewicz et al., 2014). Technofossils are the material remains of socio-technical activity, what AWG members call the ‘technosphere’ (Haff, 2014; Zalasiewicz et al., 2017), ranging from the cement foundations of buildings to plastics laid down in stratigraphic deposits, to the accumulation of chicken bones as a result of global industrialized agriculture (Bennett et al., 2018; Zalasiewicz et al., 2014). The technosphere hypothesis amounts to a strategy by which the AWG sought to expand the category of rock and fossil consistent with the established significance of palaeontology for chronostratigraphic definition. Such is the requirement of a lower boundary ‘Stratotype’ or ‘Global Boundary Stratotype Section and Point’ (GSSP), often defined by the first appearance of a novel kind of fossil (Walsh et al., 2004). Arguments such as the technofossil are a demonstration of the AWG’s effort to render arguments for a formal Anthropocene unit favourable to the ICS and International Union of Geological Sciences (IUGS), which would otherwise be met with disapproval, even indifference, as geologically irrelevant. In doing so, the category of ‘fossil’ is extended, folding geological practices of classification into socio-technical angst incorporated into the wider Anthropocene theme.

The technofossil fashions a geological temporality that accommodates the comparatively brief, seventy-year duration of the proposed Anthropocene unit within the expansive 4.5 billion-year history represented in the Geologic Time Scale. While there is no shortage of evidence that human activity has induced long-lasting changes to Earth within a short time frame, the point that the AWG found themselves having to demonstrate was that such changes were *geological*: i.e. not localized, diachronous events, but rather, an ‘isochronous array of global events that record a fundamental transition of the Earth System to a new state in which many of the parameters lie outside the range of Holocene variability’ (Waters et al., 2022, p. 2). That is what it would mean for the Anthropocene to be geologically significant, as far as the SQS are concerned. This argument had to be made with reference to testimony solicited directly from Earth itself, in keeping with the tradition of unit verification procedures particular to the SQS and ICS (Damianos, 2023). The technofossil, in other words, facilitates the articulation of an Anthropocene lower-boundary consistent with the requirements of the GSSP. The technofossil concept recruits materials previously unacknowledged in that context, such as nuclear fallout, plastics, and other novel material subsumed under the banner of the technofossil (Waters & Zalasiewicz, 2017). Whereas it has been argued (Autin & Holbrook, 2012; Finney & Edwards, 2016; Gibbard & Walker, 2013) that events associated with the Anthropocene unit are simply too recent to justify a new unit of the Chart with a level of certainty persuasive to the SQS (Gibbard et al., 2022), the premise of the technofossil demonstrates, in chronostratigraphic parlance, that there is nevertheless sufficient diversity of material evidence, in appropriate abundance, and adequately distributed around the world, to support the geological expression of a mid-twentieth century event, and consequently, the geological definition of an Anthropocene unit (Head, Zalasiewicz, et al., 2023; Head, Waters, et al., 2023; Waters et al., 2022).

## Norms are not true

The rejection of the Anthropocene raises questions and provides further opportunities for critical reflection on the production of knowledge than would have the siloing of a singularity precipitating from the term’s approval. The failed effort to formalize an Anthropocene Epoch/Series is an example of the peculiar sources of normativity in an era of climate change. The purpose of the Anthropocene was seemingly always to facilitate a normative proposition, rather than to confirm what was already known to be scientifically valid.

The fact that the Anthropocene Series/Epoch has just been rejected does not mean that anthropogenic climate change is not happening. Indeed, the failure of the Anthropocene Working Group may facilitate more nuanced understandings of the distribution of culpability and suffering deriving from climate change (which the Anthropocene has largely, even if incorrectly, become synonymous with). The method of formalization particular to amending the Geologic Time Scale, which requires a single inaugural point in the rock record (represented by a chosen stratal layer) and in time (represented by an event, in this case, the layer of ^239/240^Pu), represents a simplification and compression of the diversity of events, phenomena, and relations that characterize all that falls under the umbrella of the ‘Anthropocene’.

In its place, several geologists, some of whom were once members of the Anthropocene Working Group before resigning, proposed that the Anthropocene be recognized as an event rather than a Series/Epoch (Edwards et al., 2022; Gibbard et al., 2022). As mentioned, a Series/Epoch requires the identification of a single stratal layer and correlate event to designate the beginning of the Anthropocene. The appearance of that stratal layer must be isochronous (i.e. the layer must be of the same age) and globally dispersed (rather than confined to a single place). The AWG wanted to set the lower boundary at 1952, because various rock samples extracted from across the world indicate that rock from 1952 marks the onset of significant change in its chemical composition, primarily in terms of its content of a particular kind of plutonium from nuclear weapons detonation (McCarthy et al., 2023). Accordingly, any rock below that layer would not be ‘of the Anthropocene’. Archaeologists, anthropologists, and anyone with a passing interest in anthropogenic planetary interventions prior to the mid-twentieth century were justifiably stunned by the ignorance this gesture entailed (Bauer & Ellis, 2018; Boivin et al., 2024; Ellis et al., 2021). A geological event, by comparison, acknowledges the diachronous and multiple, simultaneous, regional episodes that collectively point to an occurrence that cannot be reduced to a singularity. The growing intensity of anthropogenic influence on planetary dynamics is acknowledged without anchoring it to a particular historical moment, without situating the Anthropocene within a single geo-anthropological narrative.

This suggests that in the era of anthropogenic climate change, science is increasingly invoked to perform social functions that have traditionally been reserved for politics and governance: facilitating standards, exercising normative power, and enforcing privilege in administrating ‘equity’. Geologists involved in the Anthropocene formalization effort invoked legal terminology, such as ‘ratifying’ units into the Geologic Time Scale. What made the Anthropocene theme interesting was not necessarily the technical detail of where the ‘lower boundary’, or ‘beginning’ was placed. Rather, it was a coupling between science and politics, in which scientific facts underpin norms. The political dimension of the SQS’s vote, according to which the AWG’s proposal was rejected on grounds of being ‘pop culture’ (Autin & Holbrook, 2012) and ‘a political decision’ (Finney & Edwards, 2016), rather than sincere science, indicates the extent to which the entire question concerning the existence of an Anthropocene unit unfolded as a debate between a group of practitioners with regards to their shared habits of knowledge construction, and the proper application of established practice.

A geoscientific attempt to define an Anthropocene has come to embroil geology, which otherwise deals with the furthest reaches of geo-history, in eminently political and normative questions of empire, nuclear warfare, and race. Measurement practices that are otherwise invoked as ‘passive’, ‘descriptive’, and ‘objective’ have come to carry new normative force. The contested nature of the Anthropocene formalization effort is an effect of the Anthropocene Working Group’s appropriating interpretive techniques, such as palaeontology and the fossil, toward a speculative account of life on Earth. In appropriating palaeontological techniques from the temporality of geological deep time to that of the present and future, members of the AWG were effectively creating a new set of tools to facilitate novel societal self-descriptions. New tools, in other words, entail new types of thinking and new practices or habits of use, which in turn entail novel self-identifications and a new geo-anthropological claims. Although the ‘technosphere’ hypothesis leaves much to be desired, it entails a provocative gesture: A new geological epoch is justifiable on the basis of a way of describing the interaction of humans and their material environments. It suggests the unfolding of a normative, anthropological observation on the register of geo-scientific fact.

## Authentic geology/stratigraphic sincerity

What does the rejection of the Anthropocene mean for geology? An interesting way to observe the significance for geoscience of the AWG’s failure is via Moeller and D’Ambrosio’s distinction between sincerity and authenticity (Moeller & D’Ambrosio, 2021). Whereas sincerity refers to the construction of identity ‘through a firm commitment of the self to its social roles’, authentic identity is ‘constructed through the creation of a social persona on the basis of one’s unique and original self’ (Moeller & D’Ambrosio, 2019, p. 575). Critical discussion of the Anthropocene theme from ‘outside’ geology has often remained indifferent to arguments occurring among geologists themselves. It has resulted in a missed opportunity to appreciate how geologists have sought to articulate normative programs on the register of geo-scientific fact, in a way that has relevance for anyone interested in the overlaps between science, law, politics, and society.

As one geologist fiercely opposed to an Anthropocene Series/Epoch put it:

[P]recise boundaries are the basis for defining geologic time, a prerequisite for the correlation of abiotic and biotic events and the understanding of the rates and timing of biological and geological processes on our planet. Earth sciences, through the International Commission on Stratigraphy of the International Union of Geological Sciences, continue to this day to define precise global boundaries, which in turn allows scientists to communicate with each other and with the public alike. (Edwards et al., 2017)

This comment makes clear the connection between a particular way of practicing and enforcing standards in geology, and the substance or possibility of communication. The above comment indicates that the practice of unit standardization is an administrative procedure that unfolds as a medium of communication. ‘This Working Group is not asking the appropriate question’, Edwards et al. (2022) elsewhere claim. They argue that the Anthropocene, when figured as a diachronous event that can accommodate a plurality of viewpoints, rather than enforcing a single layer and event in the form of an Epoch/Series, ‘serves science better’ (citation). Here, the sincerity/authenticity distinction is pertinent. Edwards et al. imply that any attempt to enforce a particular normative account of the role of a generalized and ambiguous ‘anthropos’ departs from the function of science in the provision of ‘truth’ values. For any doubt as to the normative aspirations of the AWG, consider their inheritance: an account of the Anthropocene as indicative of ‘a daunting task … for scientists and engineers to guide society towards environmentally sustainable management … [which] will require appropriate human behaviour at all scales, and may well involve internationally accepted, large-scale geo-engineering projects, for instance to ‘optimize’ climate’ (Crutzen, 2002).^2^ In entertaining Crutzen’s political opinion as a possible geological fact, rendering it material through the appropriation of the fossil as speculative device, the Anthropocene Working Group has overseen the positioning of geological observation as a technique of climate governance. In doing so, they made explicit a practice of geological observation that distinguished them from other Working Groups: a self-positioning of ingenuity and originality, applying incumbent techniques of geological observation toward normative enunciations.

Luhmann (1989) once noted, with his trademark irony, that angst is ‘the modern apriorism—not empirical but transcendental; the principle that never fails when all other principles do’ (p. 128).^3^ One of the commitments of critical theory is presumably to attend to the construction of power, of truth, and of norms as always-already contingent and partial. Yet angst resists critique. It cannot be regulated legally nor contradicted scientifically. Attempts to clarify the complex structure of risk and uncertainty only provide angst with further nourishment: Rejecting the AWG’s proposal may reinforce angst that geology is too indebted to petro-fossils to comment on climate change.

It is precisely the ambition of the Anthropocene Series/Epoch proposal that some geologists find problematic. In appropriating palaeontological techniques to advance a forward-looking account of Earth, the AWG shifted its gaze from 4.5 billion years of deep time planetary history to oracular prediction. For the most part, journalistic accounts of the Anthropocene theme took the premonitions of those geologists involved in the formalization process as a matter of scientific truth. The Anthropocene was not simply a passive observation of Earth, but was an active effort to shape a normative trajectory that journalists, policy makers, and eager scholars (myself included) could sign on to. Now that the Anthropocene Series/Epoch has been rejected, the interesting work of revealing the way in which this black box was constructed can begin.

Precisely because the Anthropocene was observed as attending to the urgency of climate catastrophe, it was popularly seen as morally justified, insofar as whoever suffers angst is morally justified. The AWG was authentic because it appropriated geology to moral and normative claims, and was morally justified in doing so vis-à-vis collective angst concerning climate change. This was to the frustration of geologists who ultimately rejected their proposal, who did so on the basis of preserving the sincerity of geological methodology (Robbins & Moore, 2013). The Anthropocene theme, therefore, unfolded as an instance of geologists observing themselves as policymakers. They became harbingers, infusing scientific communication with a morality that overlooks the distribution of culpability and suffering, seeking to set their account of the past seventy years ‘in stone’. Crucially, the AWG were able to fashion themselves as harbingers by appropriating techniques of geological observation, which traditionally furnish descriptions of the past 4.5 billion years of geological deep-time, to advance claims about how ‘humanity’ will be seen from the perspective of an undisclosed moment in the far future. The AWG could therefore be said to have been introducing an anticipatory mode of geological observation, exceeding the parameters of geology as an impartial mode of description.

## Good science’ or ‘putting the cart before the horse’

Geologists cannot know for certain that the events they describe—which often elapsed millions, if not billions, of years ago—occurred in the way they proclaim. Rather, they position themselves as intermediaries between an unknowable past and the present through established techniques of (primarily) palaeontological observation. For most, the material characteristics of an Anthropocene signature may be less significant than the fact of the signature. Yet, when geologists adopt a speculative tone, and claim impartiality in doing so by way of an allegedly ‘objective’ and ‘impartial’ interpretation of contemporary sediments, the partiality of their claims is brought into sharp relief. Geologists sought to verify their claims concerning an Anthropocene Series/Epoch by combing through precedent for amendment to the Geologic Time Scale, and positioning their proposal and communications accordingly, so as to encourage the agreement of the decision-making committees that ultimately decided against the proposed unit.

The Anthropocene formalization effort entailed a presumption of the ambiguous figure of ‘Anthropos’, which it defined by way of proposing a ‘lower boundary’ for the proposed Anthropocene Series/Epoch at sediments deposited in 1952, based on the appearance of ^239/240^Pu signals from human-made nuclear weapons detonation. Yet the AWG was arguably less concerned with a designation of the past than with a warning about the future. In doing so, members of the AWG positioned themselves as political advocates, speaking in the register of unquestionable truth rather than contestable hypothesis, paradoxically, by virtue of their scientific credentials. Here, the circumstances of the AWG come full circle, extending a tradition, or technique, of ‘science as warning’.

Paul Crutzen provides an example of normative advocacy in the guise of scientific expertise. Crutzen won the Nobel Prize for chemistry in 1995 for ‘pioneering contributions’ to understandings of ozone layer depletion, or what the press release for the award ceremony called ‘the Achilles heel of the ozone layer’ (Nobel Prize, 2024). The press release for the award announcement champions the political benefit of Crutzen’s research. Under a section entitled ‘What can we expect of the future?’ is written:

Thanks to our good scientific understanding of the ozone problem—and very largely to Crutzen [and co-winners of the Prize]—it has been possible to make far-reaching decisions on prohibiting the release of gases that destroy ozone. A protocol on the protection of the ozone layer was negotiated under the auspices of the United Nations and signed in Montreal, Canada in 1987 (Nobel Prize, 2024)

The press release adds that ‘under the latest tightening-up of the Montreal Protocol, the most dangerous gases will be totally banned from 1996’ (Nobel Prize, 2024). This was not the only instance of ‘good science’ in which Crutzen was involved. In a 1982 article entitled ‘The atmosphere after a nuclear war: Twilight at noon’, Crutzen and John Birks provide a speculative account of the immediate and long-term aftermath of ‘global nuclear war’. The article appeared in the peer-review journal *Ambio*, and comes with a frontispiece of a mushroom cloud, with the title of the article and the authors’ names imposed on top of it. The tone of the article is such that it would be at home in both a newspaper and a science journal. Indeed, the authors appear to act in several capacities simultaneously. Exhaustive descriptions of the chemical composition of atmospheric reactions resulting from nuclear weapons detonation proceed in the tone of a scientific article. Yet the text is interspersed with descriptions of how parts of the world would become unfamiliar and desolate landscapes, inducing a sense of the uncanny for Euro-American readers:

As a result of a nuclear war vast areas of forests will go up in smoke—corresponding at least to the combined land mass of Denmark, Norway and Sweden. In addition to the tremendous fires that will burn for weeks in cities and industrial centers, fires will also rage across croplands and it is likely that at least 1.5 billion tons of stored fossil fuels (mostly oil and gas) will be destroyed. The fires will produce a thick smoke layer that will drastically reduce the amount of sunlight reaching the earth’s surface. This darkness would persist for many weeks, rendering any agricultural activity in the Northern Hemisphere virtually impossible if the war takes place during the growing season. (Crutzen & Birks, 1982, p. 115)

In telling a story of how the familiar would be rendered entirely inhospitable to life as we know it, Crutzen & Birks’s piece is, literally, science fiction.

By the time of his articles on the Anthropocene, Crutzen had become more forthcoming. In concluding an article entitled ‘The geology of mankind’, authored two years after his pronouncement of the Anthropocene, Crutzen makes clear his intentions to use scientific description as a platform for normative advocacy:

Unless there is a global catastrophe—a meteorite impact, a world war or a pandemic—mankind will remain a major environmental force for many millennia. A daunting task lies ahead for scientists and engineers to guide society towards environmentally sustainable management during the era of the Anthropocene. This will require appropriate human behaviour at all scales, and may well involve internationally accepted, large-scale geo-engineering projects, for instance to ‘optimize’ climate. At this stage, however, we are still largely treading on terra incognita. (Crutzen, 2002, p. 23)

Crutzen’s advocacy for geo-engineering is well known. Somewhat ironically, for someone who sought to prevent human interference in the atmosphere, Crutzen was an early proponent of Stratospheric Aerosol Injection, which advocates releasing sulphur particles into the atmosphere to artificially induce planetary cooling through mimicry of volcanic eruptions, the effect of which is to reflect sunlight (Crutzen, 2006).

It is undeniably a fact that plutonium deposits exist in sediments around the world. The interpretation of that fact as indicative of a phenomenon called ‘the Anthropocene’ is just one of many possible ways of following on from that fact. Similarly, nothing about the advent of anthropogenic climate change justifies the kind of intervention and risk associated with geo-engineering initiatives. Crutzen’s earlier texts make for uneasy reading because they describe interventions that would inevitably affect everyone, even if they would be the result of decisions taken by a select few. Some scholars have found the ‘Anthropocene’ hypothesis unnerving for analogous reasons: a willingness to assert a unified ‘Anthropos’ and a failure to attend to the unequal distribution of culpability and suffering entailed in the phenomena described by advocates of the term (Chakrabarty, 2009). With the Anthropocene has come a culpability extended to humans as a species, without attending to racialized, gendered, and economic inequalities. This signals a radical departure from sincere ‘description of the facts’, wherein scientists are expected to toe the line to impartial observation, to a model of ‘authentic science’ (in D’Ambrosio & Moellers’s terms), whereby scientists mobilize their personal views to advocate extra-scientific claims, e.g., about ‘humanity’ as a generalized entity, as well as risk.

Yet members of the AWG continued to propose the term in the form of an ultimatum. To reject a formal Anthropocene Series/Epoch, argued AWG Chair Colin Waters, would be ‘almost akin to someone saying climate change doesn’t exist’ (Ley, 2023). Clearly that is not true. Yet in asserting such claims in the register of geo-scientific fact, AWG members sought to immunize their questionable assertions from critique, concealing the extent to which the claim was a partial description.

For Waters, the proposal for a formal Anthropocene Series/Epoch should be accepted, if not on the basis of scientific rigour, or agreement with previous decisions concerning amendments to the Geologic Time Scale, then at the very least out of a moral obligation to acknowledge anthropogenic climate change. Framing such an assertion as an impartial, geo-scientific description positions the claim as simply a scientific truth, and therefore incontestable, rather than as supporting norms. Furthermore, the fact that the AWG proposed a Series/Epoch that would not simply last the past seventy years, but would extend into the future, meant that any description or definition of the Anthropocene Series/Epoch was also at once a warning about what lay just beyond the horizon, and an imperative to act accordingly in the present to avert all that such a threat entails. Precisely because this warning was framed as scientific truth, it transformed the uncertainty of the prophecy into the certainty of a warning: the certainty of a sense of concern or angst about the future that is yet to come. Waters presents the pending decision on the Anthropocene as a duty to worry, and so support of its formalization as morally justified—even if the future that Waters’s angst presumes does not materialize. Angst does not need a theoretical foundation, because those who articulate their angst can be justified in the certainty of their concern for the future, even if it turns out to be unjustified, or false. It is for this reason that Luhmann explains that, with regard to ecological communications, or communications about the certainty of uncertainty about a collective ecological future, angst has come to replace reason as the unassailable *a priori*. ‘Angst resists any critique of pure reason’, he explains: ‘It is the modern apriorism—not empirical but transcendental; the principle that never fails when all other principles do’ (Luhmann, 1989, p. 130).

As a consequence of infusing reason with angst, or description with warning, geology, according to the AWG, assumes qualities more traditionally associated with the news cycles of mass-media reportage. At the 2023 meeting of the International Commission on Stratigraphy, there were almost as many journalists present as there were stratigraphers and geologists. The journalists were not there to cover any topic other than the Anthropocene. On July 8th, 2023, three days before the 4th Annual Congress of the International Commission on Stratigraphy commenced, the news outlet Barron’s already included an announcement of the Anthropocene Working Group, at the conference, in its ‘International 7-day News Agenda’: ‘(*) LILLE (France)—Announcement of the site selected to embody the Anthropocene epoch by the Anthropocene Working Group (1700 GMT) VIDEO’. My own request to attend the conference as a participant observer was met with scepticism by the conference organizer. Some explanation as to my intentions was requested. Upon satisfactory provision of those details, the conference organizer revealed their opinion concerning the Anthropocene formalization effort as it has unfolded within stratigraphy: ‘It is surprising that a few former palaeontologists and stratigraphers left discipline [sic], the poorly impact-related research on Palaeozoic fossils, or Quaternary microfossils, to focus on this subject. Was it to finally get some more media attention?’ The conference organizer continued: ‘Research, geology and palaeontology, is more and more populistic. Our discipline is “*Nature*-driven”, at least in the UK, where the research assessment system seem to count very much on impact and impact factors. Only few subjects make it to the mass media. Are those who work on these subjects the better scientists?’^4^

**Figure 2.**
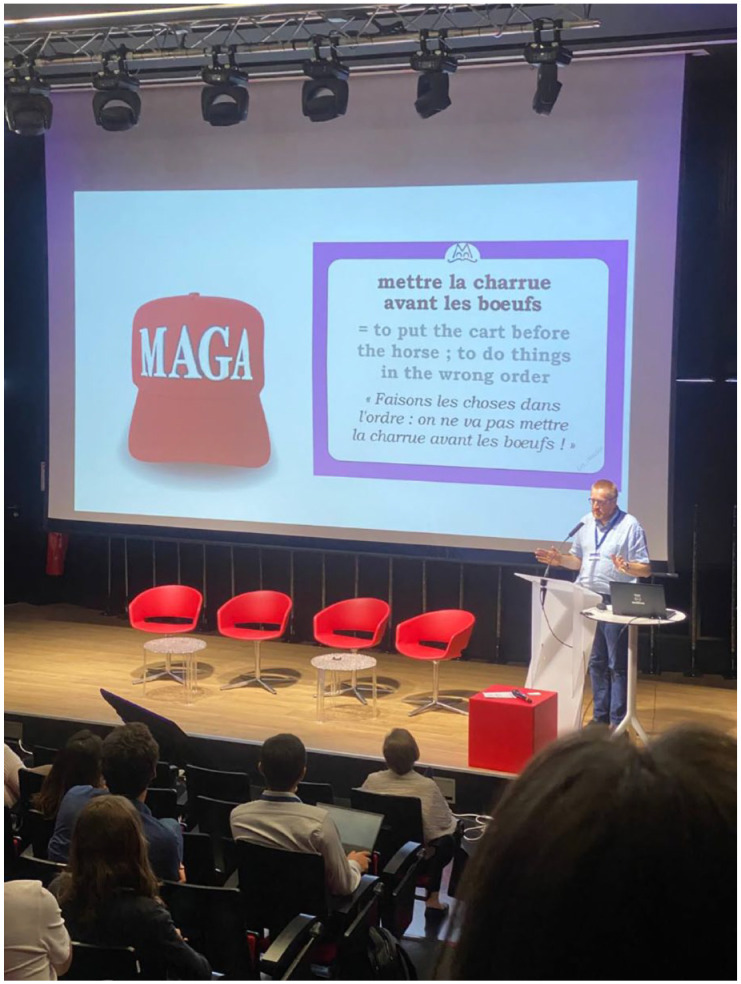
Thomas Servais delivers the closing ceremony of the 4th Annual Congress of the International Commission on Stratigraphy, on the 13th of July, 2023, in Lille, France. MAGA, in this context, is alleged to stand for ‘Make the Anthropocene great again’. *Source:* Photograph taken by author on 13 July, 2023.

The conference organizer’s positioning of the Anthropocene as a mass-media phenomenon, rather than a scientific one, was telling. Furthermore, it was accurate, insofar as journalistic interest in the event by virtue of the media announcement of an AWG announcement became a persistent theme over the following few days. The journalists were forbidden from entering the venue; according to the organizers, they had not obtained the appropriate license to film conference proceedings. However, several camera and sound crews waited just beyond the sliding doors of the conference hall. AWG members took turns between panel sessions to go outside and speak to cameras, receiving directions from camera crews to walk in certain directions on camera, and ‘speak about something’ while being filmed, to fill in news segments between cuts of interviews (Figure 2).

On the last day of the congress, during the ‘closing ceremony’, proceedings took an unusual turn. During the conference organizer’s closing remarks, they produced a slide displaying a red MAGA hat. MAGA, in this context, is alleged to stand for ‘Make the Anthropocene great again’. To the side, read: ‘mettre la charrue avant les boeufs = to put the cart before the horse; to do things in the wrong order’. This jarring provocation was a response to a decision made by the AWG to host offsite, at a hotel, fifteen minutes’ walk from the congress’, a formal announcement of the chosen ‘lower boundary’ candidate (GSSP) for the proposed Anthropocene Series/Epoch. In front of several camera crews, including a live-stream into the Berlin-based Haus der Kulturen der Welt (which funded the AWG’s core extraction process, which is a story for another time), AWG Chair Colin Waters announced, pausing for suspense, that the GSSP candidate for the Anthropocene Series/Epoch would be in Crawford Lake, Canada. The cart came before the horse: An announcement had been made as if the GSSP had been decided, when it had yet to be formally approved by the decision-making committee of the SQS and ICS. The hastiness of the AWG was perceived as an attempt to subvert the procedures associated with amendments to the Geologic Time Scale through mass-media news. If enough journalists could report on the AWG’s effort, then maybe that could provoke the decision-making committees into formal approval, lest they be perceived as either disorganized or, worse, ethically compromised.

Luhmann’s insights concerning the anxious *a priori* are revealing in this regard. They suggest that the AWG’s formalization effort proceeded as an attempt to minimize the difference between the scientific function of truth claims and the mass-media function of procuring novelty and sensation. When Waters posits the formalization effort as an ultimatum, whereby the SQS either recognizes climate change or denounces it, the register of the decision-making committee’s remit is shifted from one of verifying a scientific observation to supporting a normative assertion. Waters’s comment would suggest that the SQS was under a moral obligation to accept the AWG’s proposal, not exclusively on the basis of the merit of their report, but on the basis of a shared acknowledgement of anthropogenic climate change, and the urgency of acting on it.

Waters is an authentic geologist, as described by Moeller and D’Ambrosio. Leading the AWG, Waters advocates a novel appropriation of geoscientific methods toward the kinds of articulations that characterized Crutzen’s use of science as a platform for normative advocacy. While journalists were keen to ‘get the scoop’ on the Anthropocene, the AWG, too, responded to media interest in their work to provoke a decision concerning the geological title of the contemporary, side-stepping the formal procedures established to do so. Whereas sincere geology would assert the primacy of decision-making procedures that have been established over generations, authentic geology practices a coupling of scientific truth claims with normative advocacy and mass-media news cycles, such that the object of the AWG was not so much the past seventy years of deposits, but the administration of geological time and space itself. The AWG, in other words, articulated a second-order observation of geological time. Its descriptions of strata, even while grounded in analogies to earlier decisions, amounted to a redescription of what could count as strata, or legitimate geological temporalities.

Members of the AWG acted in such a way that they clearly perceived their own work as contributing to far more than the classification of geological time. In what capacity did they believed themselves to be acting? Why did they feel that geological knowledge was an appropriate forum to advance speculative and normative claims. In this instance, the Geologic Time Scale becomes much more than a simple technique of nomenclature relevant to geo-scientists. It is an instrument with which to steer society more generally, insofar as it provides a space with which to justify a warning about the future, which—a normative claim—in the register of geo-scientific fact. This caused significant concern among stratigraphers, such as those present at the 4th Annual Congress of the International Commission on Stratigraphy. And it is noteworthy that the AWG sought to refute the priority of the decision-making procedures those bodies have established, by developing close ties with journalists and mass-media outlets, in an attempt to pre-determine the outcome of the Anthropocene vote ‘in the court of public opinion’.

The AWG’s gesture attempts to link scientific truth with the mechanisms of mass-media sensation and political normativity. While Crutzen was an early practitioner of this genre of coupling, the Anthropocene formalization effort suggests that this is a gesture that will continue as long as there is a perceived absence of adequate responses from traditional normative avenues in response to the perceived ‘climate crisis’. While this is an argument made many times before (Hulme, 2009; Jasanoff, 2005; McKittrick, 2021; Oreskes & Conway, 2012), the ethnographic experience I recount in this article provides an ethnographic account of coupling in practice.

The authenticity/sincerity thematic, as articulated by Moeller & D’Ambrosio, is thoroughly a mass-media phenomenon. Mass-media provides a space where observers can observe themselves being observed, positioning themselves in accordance with how they want to be observed observing, or how they want an issue to be received, in such a way that they can create that issue anew through intentional selection (or curation) of the relevant factors that the receiving observers should bear in mind. The AWG’s formalization effort unfolded as a subversion of the established procedures of the ICS, drawing on mass-media attention to frame the decision-making procedure in a way that benefited the AWG’s intentions, allowing its members to position themselves as morally justified, and therefore, controversially, as scientifically legitimate. Members of the AWG are therefore self-assertively virtuous, at least as their own self-presentation is concerned, even if the claim they were asserting has been rendered, through popular vote, scientifically false. The consequence of the AWG’s coupling of science, normativity, and media sensation is that scientific truth might be increasingly synonymous with moral legitimacy, even though the latter is necessarily contingent on the ability of whoever claims moral authority to convince others that their ethical vision is ‘just’.

## Notes

1. The AWG received one million euros from the Haus der Kulturen der Welt in Berlin, which is an important feature of the formalization effort into which I shall not go here, for the sake of brevity.

2. I call this their ‘inheritance’ insofar as it was on the basis of Crutzen’s (2002) initial article defining the Anthropocene term that was the impetus for the commission of the Anthropocene Working Group.

3. The term angst appears in the original German edition of Okologische Kommunikation (Luhmann, 2008). It has been translated into the English edition as anxiety. However, angst refers to a combination of fear and anxiety. Whereas anxiety may refer to anticipation, even excitement, angst designates an element of fear that is more appropriate to reflection concerning the unfolding of anthropogenic climate change.

4. Personal correspondence. The term ‘Nature-driven’ here refers to the journal Nature.
